# Shear Elasticity of Magnetic Gels with Internal Structures

**DOI:** 10.3390/s18072054

**Published:** 2018-06-27

**Authors:** Dmitry Borin, Dmitri Chirikov, Andrey Zubarev

**Affiliations:** 1 Chair of Magnetofluiddynamics, Measuring and Automation Technology, TU Dresden, 01069 Dresden, Germany; dmitry.borin@tu-dresden.de; 2 Department of Theoretical and Mathematical Physics, Ural Federal University, Lenina Ave 51, 620083 Ekaterinburg, Russia; d.n.chirikov@urfu.ru; 3 M.N. Mikheev Institute of Metal Physics of the Ural Branch of the Russian Academy of Sciences, Sofia Kovalevskaya st., 18, 620219 Ekaterinburg, Russia

**Keywords:** magnetic gels, shear modulus, chain structures, magnetorheological effect

## Abstract

We present the results of the theoretical modeling of the elastic shear properties of a magnetic gel, consisting of soft matrix and embedded, fine magnetizable particles, which are united in linear chain-like structures. We suppose that the composite is placed in a magnetic field, perpendicular to the direction of the sample shear. Our results show that the field can significantly enhance the mechanical rigidity of the soft composite. Theoretical results are in quantitative agreement with the experiments.

## 1. Introduction

Magnetic gels are composites of nano- and micron-sized magnetic particles in soft polymer matrixes. The combination of a rich set of physical properties of the polymer and magnetic materials is very valuable for many progressive industrial, bioengineering, and biomedical applications [1,2,3,4,5,6,7]. In part, for address drug delivery, for industrial and biological sensors [8,9,10,11,12,13,14], for the construction of soft actuators and artificial muscles [2,15], and for regenerative medicine and tissue engineering [16,17,18,19,20,21,22,23,24,25,26,27,28,29,30,31,32,33,34,35,36,37,38,39,40,41]. An overview of the works on magnetic gel synthesis and their biomedical applications can be found in [23].

One of the remarkable properties of magnetic gels is their ability to change, under the action of an external magnetic field, their microstructure, magnetic, mechanical, and other microscopic properties, size, and shape. This provides the opportunity to control, with the help of the field, mechanic behavior, transport, and electrical processes in these systems, and this possibility presents a significant advantage for biosensoric, tissue engineering, and other biological applications [8,9,20,23,39,41].

During the magnetic gels’ synthesis, the particles are usually embedded in the liquid polymer, and their spatial distribution is fixed after the composite gelation. If the host polymer is cured without an external magnetic field, the particles, as a rule, are distributed more or less homogeneously and isotropically. If the composite is polymerized under the field (field of polymerization), the particles form various anisotropic structures, elongated in the field direction. The appearance of these internal structures significantly changes the sensitivity of the gels to mechanic, electrical, magnetic, and other external impacts, and changes the kinetics of the internal transport phenomena and chemical reactions, the rate of cell proliferation, and other phenomena in these systems. This opens perspectives of the tunable synthesis of the magnetically controlled sensors, and scaffolds for the growth of cell tissues with willing structure and properties, artificial muscles, and other materials for biological and industrial applications.

The simplest kind of the internal structures that are formed by the magnetic particles in liquid media are liner chains, where particles are bounded as “head to tails” by the forces of magnetic attraction. These structures appear in the systems with a low and moderate volume concentrations of magnetic particles (usually in the range of 10–15%). Some photos of these chains can be found, for example in [42,43]. In the case of the higher concentrations, the particles can form topologically more complicated branched, net-like, bulk and other structures (see, for example, [44,45,46,47,48,49,50]).

The effect of the magnetic field on the elastic shear modulus of the gels with a homogeneous and isotropic (gas-like) spatial distribution of magnetic particles has been theoretically studied in refs. [51,52]. It was shown that a magnetic field that is applied perpendicular to the macroscopic shear of the composite, enhances the composite elastic modulus. At the same time, the experiments [53] show that the rigidity of the composites with the chains aligned perpendicularly to the shear, is significantly more than that of the systems with a chaotic distribution of the particles. Since the micromechanic (on the level of the particles and their aggregates) situation in the composites with the heterogeneous aggregates is significantly different from that in the homogeneous systems, the microscopic analysis of macroscopic properties of the composites with internal heterogeneous structures requires the development of a special theoretical approach.

The aim of this work is to theoretically study the effect of an external magnetic field on the shear elastic modulus of magnetic gels with internal chain-like structures, that are formed by magnetizable non-Brownian spherical particles. Physically, this means that the size of the particles are supposed to range from several tens of nanometers to microns. The particles of this size are very often used for the preparation of magnetic gels for bio-medical applications.

We take into account that the chains appear at the stage of the matrix polymerization under the action of an external magnetic field. That is why all chains are parallel to this field. We suppose that the actual magnetic field **H** has the same direction as the field of polimerization, i.e., that the field **H** is parallel to the chains. We consider the situation when the macroscopic shear of the sample is perpendicular, whereas the gradient of the shear is parallel to the chains. The length of the chains is supposed to be much less than the size of the sample.

## 2. Physical and Mathematical Model

**Figure 1:**
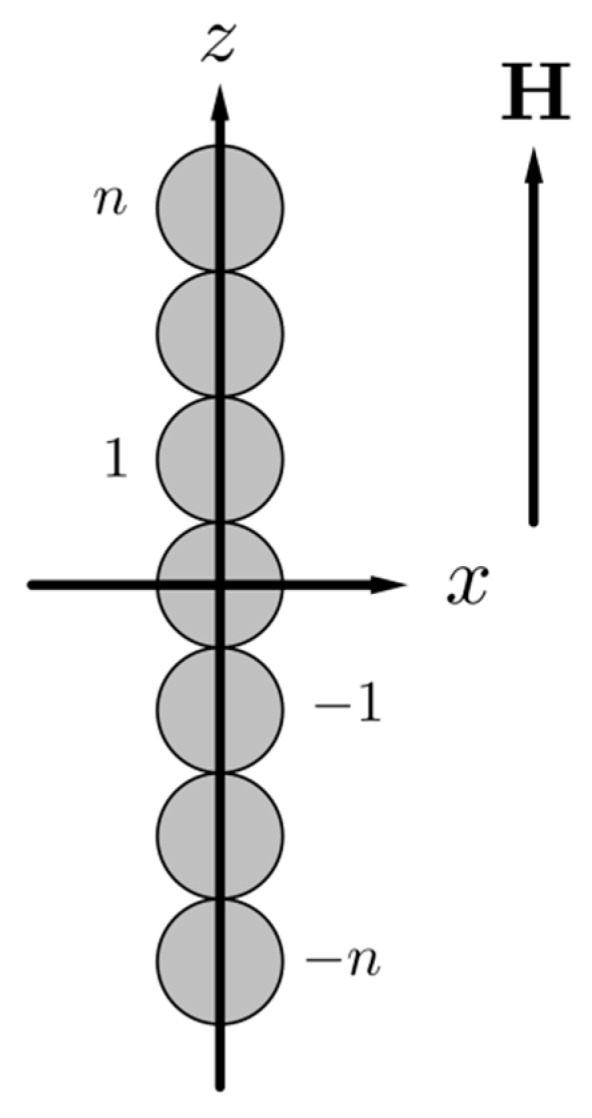
An illustration of the chain model and the used Cartesian coordinate system; *n* is the number of particles at the extremity of the chain, starting from the central one, which is number 0.

For maximal simplification of the mathematical part of the problem, we will suppose that the particles are identical. Like in [54,55], we will neglect fluctuations of the chain’s shape and will consider them as ideally straight aggregates, aligned along the applied magnetic field **H**. This model of the chain is illustrated in Figure 1.

The typical size of the cell of the polymer matrix in ferrogels is several nanometers, and the size of the particles vary from several tens of nanometers to microns. Thus, the particles are much larger than the gel cell. That is why we will consider the host polymer as a continuous medium with respect to the particles.

We will restrict ourselves by the analysis of small deformations of the composites and will suppose the linear relations between the mechanic stress and deformations in the matrix.

We will also neglect any interactions between the chains. This approximation is based on the results of ref. [56], which shows that the effects inside the chains play a dominant role in the formation of macroscopic properties of the composites, compared with the effects of the interchain interaction.

For mathematical definiteness, we will suppose that the chain consists of an odd number of particles. This assumption is not of principle for the physical analysis.

**Figure 2:**
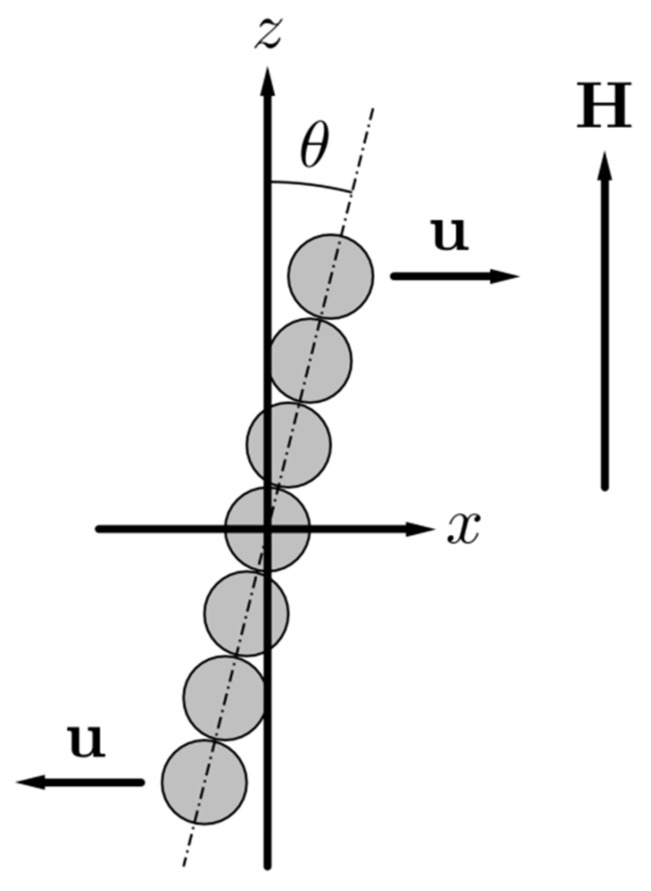
Illustration of the shearing of the chain.

Let us denote the mean vector of a material point displacement in the composite as **u**. In the coordinate system, shown in Figure 2, the vector **u** is the mean displacement in the composite (i.e., the displacement at the infinitive distance from the chain). The vector **u** can be presented as: $u_{x}=\gamma x$ where *γ* is the mean shear of the system. We will consider small shear deformations of the composite, which means that the strong inequality γ≪1 is held.

Let the total number of the particles *N* in a chain be N=2n+1, where *n* is an integer. In the framework of the used approximations, the equations of the stationary displacement of the particles in the chain can be presented in the following form [54]:

(1) $$3\pi G_{0}d\left(\gamma id-u_{i}\right)+f_{i}^{\left(m\right)}=0,\;0<i\leq n,\;-n\leq i<0,\;u_{0}=0.$$

Here, *G*_0_ is the shear modulus of the matrix, *d* is the diameter of the particles, $f_{i}^{\left(m\right)}$ is the force of the magnetic interaction of the *i*-th particle with the neighboring particles of the chain, and for the central particle i=0. The equality $u_{i}=-u_{-i}$ for 0<i≤n follows from the symmetry of the problem.

For convenience, we introduce the dimensionless magnetic force ${\tilde{f}}_{i}^{\left(m\right)}$ and dimensionless displacement ${\tilde{u}}_{i}$:

(2) $${\tilde{f}}_{i}^{\left(m\right)}=\frac{f_{i}^{\left(m\right)}}{\pi G_{0}d^{2}},\;{\tilde{u}}_{i}=\frac{u_{i}}{d},\;0\leq i\leq n.$$

By using these notations, one can rewrite Equation (1) as:

(3) $$3\left(\gamma i-{\tilde{u}}_{i}\right)+{\tilde{f}}_{i}^{\left(m\right)}=0,\;{\tilde{u}}_{i}={\tilde{u}}_{-i},\;0<i\leq n,\;{\tilde{u}}_{0}=0.$$

Under the assumption that γ≪1, the inequality ${\tilde{u}}_{i}\ll 1$ is held.

The magnetic force ${\tilde{f}}_{i}^{\left(m\right)}$ of the interparticle interaction can be estimated in the framework of the simplest dipole-dipole approximation. In order to calculate ${\tilde{f}}_{i}^{\left(m\right)}$, we need to determine the magnetic moments of the particles in the chain. Strictly speaking, the value of a particle moment depends on the number *i* of the particle position in the chain. However, analysis [55] shows that approximation, where magnetic moments of all of the particles in the chain are supposed identical, leads to not significant deviations from the strict approach. That is why we will use the simplest approximation of identity of the particle moments in the chain.

When the composite experiences the macroscopic shear deformation, the axis of the chain deviates from the *z*-axis, as is illustrated in Figure 2. Because of the mutual magnetization of particles, the vector of the particle magnetic moment will also be deviated from this axis. Therefore, both components *M_x_* and *M_z_* of the vector **M** of the particle magnetization will take place in the deformed composite.

We estimate the magnetic force ${\tilde{f}}_{i}^{\left(m\right)}$ by using the nearest-neighbor approximation, taking into account the magnetic dipole-dipole interaction only between the neighbor particles in the chain. By using the well-known relation for the force of the dipole-dipole interaction (see, for example, [57]), after simple but cumbersome transformations, in the linear approximation with respect to the displacements ${\tilde{u}}_{i}$, one can get:

(4) $${\tilde{f}}_{1}^{\left(m\right)}={\tilde{f}}_{1,0}^{\left(m\right)}+{\tilde{f}}_{1,2}^{\left(m\right)}=\frac{\beta {\tilde{M}}_{z}{\tilde{M}}_{x}}{24}-\frac{\beta {\tilde{M}}_{z}^{2}{\tilde{u}}_{1}}{12}-\left[\frac{\beta {\tilde{M}}_{z}{\tilde{M}}_{x}}{24}+\frac{\beta {\tilde{M}}_{z}^{2}\left({\tilde{u}}_{1}-{\tilde{u}}_{2}\right)}{12}\right]\;=\frac{\beta {\tilde{M}}_{z}^{2}\left({\tilde{u}}_{2}-2{\tilde{u}}_{1}\right)}{12},\;\;{\tilde{f}}_{i}^{\left(m\right)}={\tilde{f}}_{i,i-1}^{\left(m\right)}+{\tilde{f}}_{i,i+1}^{\left(m\right)}=\frac{\beta {\tilde{M}}_{z}{\tilde{M}}_{x}}{24}+\frac{\beta {\tilde{M}}_{z}^{2}\left({\tilde{u}}_{i-1}-{\tilde{u}}_{i}\right)}{12}-\left[\frac{\beta {\tilde{M}}_{z}{\tilde{M}}_{x}}{24}+\frac{\beta {\tilde{M}}_{z}^{2}\left({\tilde{u}}_{i}-{\tilde{u}}_{i+1}\right)}{12}\right]\;\;=\frac{\beta {\tilde{M}}_{z}^{2}\left({\tilde{u}}_{i+1}-2{\tilde{u}}_{i}+{\tilde{u}}_{i-1}\right)}{12},\;1<i<n,\;\;{\tilde{f}}_{n}^{\left(m\right)}={\tilde{f}}_{n,n-1}^{\left(m\right)}=\frac{\beta {\tilde{M}}_{x}{\tilde{M}}_{z}}{24}+\frac{\beta {\tilde{M}}_{z}^{2}\left({\tilde{u}}_{n-1}-{\tilde{u}}_{n}\right)}{12},\;{\tilde{M}}_{x}=\frac{M_{x}}{M_{s}},\;{\tilde{M}}_{z}=\frac{M_{z}}{M_{s}},\;\beta =\frac{\mu_{0}M_{s}^{2}}{G_{0}}.$$

Here, $f_{i,i\pm 1}^{\left(m\right)}$ is the force of the magnetic interaction between the *i*-th particle in the chain with the neighbor particle, *M_s_* is the saturated magnetization of the particle’s material, *β* is the parameter which defines the ratio of the energy of magnetic interaction between two magnetically saturated particles to the energy of elastic deformation of the matrix, and *μ*_0_ is the vacuum magnetic permeability.

The main problem now is to estimate the dimensionless components ${\tilde{M}}_{x}$ and ${\tilde{M}}_{z}.$ The strict solution of a problem of determination of magnetic moments of two closely situated magnetizable particles has not been obtained in literature because of not overcoming mathematical complexity. Here, we use the approach [58], where each particle is considered to be situated in a uniform magnetic field $H^{\left(e\right)}$, consisting of the external, with respect to the particle’s field **H** (i.e., the mean field in the sample), and the field created by the other particle in the center of the first one. It is supposed that the magnetization of the particle obeys to the nonlinear Frolich-Kennelly relation [59]:

(5) $${\tilde{M}}_{k}=\frac{\chi_{p}{\tilde{H}}_{k}^{\left(i\right)}}{1+\chi_{p}{\tilde{H}}^{\left(i\right)}},\;k=x,z,\;{\tilde{H}}^{\left(i\right)}=\sqrt{{\left({\tilde{H}}_{x}^{\left(i\right)}\right)}^{2}+{\left({\tilde{H}}_{z}^{\left(i\right)}\right)}^{2}}.$$

Here, $\chi_{p}$ is the initial magnetic susceptibility of the particle material, and $H^{\left(i\right)}$ is the magnetic field inside the particle. For the spherical particle the last field, this can be found from the general relation [60]:

(6) $${\tilde{H}}_{k}^{\left(i\right)}+\frac{{\tilde{M}}_{k}}{3}={\tilde{H}}_{k}^{\left(e\right)},\;k=x,z.$$

Taking into account the magnetic interaction between the neighbor particles in the chain, in the dipole-dipole approximations we get:

(7) $${\tilde{H}}_{x}^{\left(e\right)}=\frac{3{\tilde{M}}_{z}\Psi_{n}^{\left(1\right)}-{\tilde{M}}_{x}\Psi_{n}^{\left(0\right)}}{12N},\;{\tilde{H}}_{z}^{\left(e\right)}=\tilde{H}+\frac{3{\tilde{M}}_{x}\Psi_{n}^{\left(1\right)}+2{\tilde{M}}_{z}\Psi_{n}^{\left(0\right)}}{12N}.\;\Psi_{n}^{\left(0\right)}=\overset{2n}{\underset{i=1}{\sum }}\frac{2n+1-i}{i^{3}},\;\Psi_{n}^{\left(1\right)}=2\left(a_{n-1}{\tilde{u}}_{n-1}+a_{n}{\tilde{u}}_{n}\right),\;a_{i}=\overset{n+i}{\underset{j=n+1-i}{\sum }}\frac{1}{j^{4}}.$$

Combining Equations (5)–(7) and Notations (4), after some transformations we come to the following system of nonlinear algebraic equations with respect to the dimensionless components ${\tilde{M}}_{x}$ and ${\tilde{M}}_{z}$ of the particle magnetization:

(8) $$\;\frac{{\tilde{M}}_{x}}{\chi_{p}\left(1-\sqrt{{\tilde{M}}_{x}^{2}+{\tilde{M}}_{z}^{2}}\right)}+C_{x}{\tilde{M}}_{x}-A{\tilde{M}}_{z}=0,\;\frac{{\tilde{M}}_{z}}{\chi_{p}\left(1-\sqrt{{\tilde{M}}_{x}^{2}+{\tilde{M}}_{z}^{2}}\right)}+C_{z}{\tilde{M}}_{z}-A{\tilde{M}}_{x}-\tilde{H}=0,\;\;C_{z}=\frac{1}{3}\left(1-\frac{\Psi_{n}^{\left(0\right)}}{2N}\right),\;C_{x}=\frac{1}{3}\left(1+\frac{\Psi_{n}^{\left(0\right)}}{4N}\right),\;A=\frac{\Psi_{n}^{\left(1\right)}}{4N},$$

This system can be solved analytically only under condition ${\tilde{M}}_{x}\ll {\tilde{M}}_{z}.$ The last is true when the sample deformation is small (i.e., when γ≪1). By using the linear approximation with respect to ${\tilde{M}}_{x}$, one gets from (8):

(9) $${\tilde{M}}_{z}=\frac{D_{z}-\sqrt{D_{z}^{2}-4C_{z}\tilde{H}}}{2C_{z}},\;{\tilde{M}}_{x}=\frac{{\tilde{M}}_{z}\left(a_{n-1}{\tilde{u}}_{n-1}+a_{n}{\tilde{u}}_{n}\right)}{2NE},\;\;D_{z}=\frac{1}{\chi_{p}}+C_{z}+\tilde{H},\;D_{x}=\frac{1}{\chi_{p}}+C_{x}+\tilde{H},\;E=D_{x}-C_{z}{\tilde{M}}_{z}.$$

Parameter $a_{n}$ can be found from the definition of $a_{i}$ in Equation (7). Let us remind that the result (9) is obtained in the approximation [55] of the identity of magnetic moments and therefore, of is the identity of magnetization **M** of all of the particles in the chain.

Combining the relations (3), (4), and (9), we come to the system of the linear algebraic equations with respect to the dimensionless displacement ${\tilde{u}}_{i}$:

(10) $$2\left(18+\beta {\tilde{M}}_{z}^{2}\right){\tilde{u}}_{1}-\beta {\tilde{M}}_{z}^{2}{\tilde{u}}_{2}=36\gamma ,\;-\beta {\tilde{M}}_{z}^{2}{\tilde{u}}_{i-1}+2\left(18+\beta {\tilde{M}}_{z}^{2}\right){\tilde{u}}_{i}-\beta {\tilde{M}}_{z}^{2}{\tilde{u}}_{i+1}=36\gamma i,\;1<i<n,\;-\beta {\tilde{M}}_{z}^{2}\left(a_{n-1}+4NE\right){\tilde{u}}_{n-1}+\left[4NE\left(36+\beta {\tilde{M}}_{z}^{2}\right)-\beta {\tilde{M}}_{z}^{2}a_{n}\right]{\tilde{u}}_{n}=144\gamma nNE.$$

This system can be solved either analytically, or numerically.

The total shear stress *σ* in the composite that is placed in magnetic field can be presented as:

(11) $$\;\sigma =\sigma^{\left(0\right)}+\sigma^{\left(m\right)}.$$

Here, $\sigma^{\left(0\right)}$ and $\sigma^{\left(m\right)}$ are the nonmagnetic and magnetic parts of the stress that are produced by the aggregates. The nonmagnetic part of the stress appears because of the local inhomogeneous deformations of the elastic matrix, caused by the presence of the chains.

Nowadays, there is no a strict theoretical description of the elastic interaction of the chain with the environment. That is why we estimate $\sigma^{\left(0\right)}$ by using the approach [61], modeling the *N*-particles chain as a prolate ellipsoid of revolution with the minor and major axes equal to the particle diameter *d* and *Nd*, respectively. It is of fundamental importance that the volume of this ellipsoid is equal to the total volume of all of the particles in the chain. Therefore, the volume concentration of these ellipsoids is equal to the volume concentration of the particles in the ferrogels.

By using the results of the mechanics of suspensions of ellipsoidal particles [62], in the linear approximation with respect to the shear, we get:

(12) $$\sigma^{\left(0\right)}=G_{0}\gamma +G_{0}\varphi \gamma \underset{N}{\sum }\left[\alpha_{N}+\frac{\zeta_{N}+\beta_{N}\left(1+\lambda_{N}\right)}{2}\right]F_{N}.$$

Here, *φ* is the volume concentration of the particles; *α_N_*, *β_N_*, *λ_N_*, and *ζ_N_* are some coefficients, which depend on the length of the chain. The explicit forms of these coefficients are given in the Appendix A; $F_{N}$ is a function of distribution over the number *N* of the particles in the chains, normalized so that $\sum_{N}F_{N}$ = 1. This function depends on many factors and features of the composite synthesis (size and concentration of the particles, viscosity and kinetics of the host polymer curing, the strength of the field of polymerization, etc.). The determination of the function $F_{N}$ presents a separate problem. Theoretical study of evolution over time of this function in the magnetic suspensions with a permanent viscosity of the currier liquid has been done in [63]. This model is based on the analysis of a system of the Smoluchowski equations, which describes the kinetic of the aggregation of the chains with a various number of particles.

In the case of cured magnetic gel, this evolution must be studied by taking into account change and time of the rheological properties of the host polymer. Depending on the molecular structure of the polymer, the concentration, and the chemical properties of the curing agent, these properties of evolution can obey to different laws, which can hardly be presented in a general form. That is why here, we suppose that the function $F_{N}$ is known from either independent experiments or theoretical analysis.

By using the results [64,65] for the macroscopic stress in a system of chain-like polymer macromolecules, we get the following estimate for the magnetic stress $\sigma^{\left(m\right)}$:

(13) $$\;\sigma^{\left(m\right)}=-\frac{2\varphi }{v_{p}}\underset{N}{\sum }\frac{1}{N}\left[\overset{n}{\underset{i=1}{\sum }}f_{i,i-1}^{\left(m\right)}d\right]F_{N}.$$

Here, $v_{p}$ is the volume of the particle.

By definition, the shear modulus of the composite *G*:

(14) $$G=\frac{\sigma }{\gamma }.$$

Let us introduce the dimensionless stresses:

(15) $$\tilde{\sigma }=\frac{\sigma }{G_{0}},\;{\tilde{\sigma }}^{\left(0\right)}=\frac{\sigma^{\left(0\right)}}{G_{0}},\;{\tilde{\sigma }}^{\left(m\right)}=\frac{\sigma^{\left(m\right)}}{G_{0}},\;\tilde{\sigma }={\tilde{\sigma }}^{\left(0\right)}+{\tilde{\sigma }}^{\left(m\right)},\;{\tilde{\sigma }}^{\left(0\right)}=\gamma +\varphi \gamma \underset{N}{\sum }\left[\alpha_{N}+\frac{\zeta_{N}+\beta_{N}\left(1+\lambda_{N}\right)}{2}\right]F_{N},\;{\tilde{\sigma }}^{\left(m\right)}=-12\varphi \underset{N}{\sum }\frac{1}{N}\left[\overset{n}{\underset{i=1}{\sum }}{\tilde{f}}_{i,i-1}^{\left(m\right)}\right]F_{N}=-12\varphi \underset{N}{\sum }\frac{1}{N}\overset{n}{\underset{i=1}{\sum }}\left[\frac{\beta {\tilde{M}}_{z}{\tilde{M}}_{x}}{24}+\frac{\beta {\tilde{M}}_{z}^{2}\left({\tilde{u}}_{i-1}-{\tilde{u}}_{i}\right)}{12}\right]F_{N}\;=\varphi \beta \underset{N}{\sum }\frac{{\tilde{M}}_{z}^{2}}{N}\left[{\tilde{u}}_{n}-\frac{n\left(a_{n-1}{\tilde{u}}_{n-1}+a_{n}{\tilde{u}}_{n}\right)}{4NE}\right]F_{N},$$

and the dimensionless shear modulus of the composite $\tilde{G}$:

(16) $$\;\tilde{G}=\frac{G}{G_{0}}=\frac{\tilde{\sigma }}{\gamma }.$$

The shear modulus ${\tilde{G}}^{\left(0\right)}$ of the composite without a magnetic field can be found from Equation (12):

(17) $$\;{\tilde{G}}^{\left(0\right)}=\frac{{\tilde{\sigma }}^{\left(0\right)}}{\gamma }=1+\varphi \underset{N}{\sum }\left[\alpha_{N}+\frac{\zeta_{N}+\beta_{N}\left(1+\lambda_{N}\right)}{2}\right]F_{N}.$$

The magnetically induced part $\Delta \tilde{G}$ of the shear modulus can be calculated as (14):

(18) $$\Delta \tilde{G}=\tilde{G}-{\tilde{G}}^{\left(0\right)}=\frac{{\tilde{\sigma }}^{\left(m\right)}}{\gamma }=\frac{\varphi \beta }{4E}\underset{N}{\sum }\frac{{\tilde{M}}_{z}^{2}}{N^{2}}\left[\left(4NE-na_{n}\right)x_{n}-na_{n-1}x_{n-1}\right]F_{N},\;x_{i}=\frac{{\tilde{u}}_{i}}{\gamma }.$$

For the chain with each number *N* of the particles, the dimensionless displacements *x_i_* are determined from the system of Equation (10), which can be presented in the form:

(19) $$2\left(18+\beta {\tilde{M}}_{z}^{2}\right)x_{1}-\beta {\tilde{M}}_{z}^{2}x_{2}=36,\;-\beta {\tilde{M}}_{z}^{2}x_{i-1}+2\left(18+\beta {\tilde{M}}_{z}^{2}\right)x_{i}-\beta {\tilde{M}}_{z}^{2}x_{i+1}=36i,\;1<i<n,\;-\beta {\tilde{M}}_{z}^{2}\left(a_{n-1}+4NE\right)x_{n-1}+\left[4NE\left(36+\beta {\tilde{M}}_{z}^{2}\right)-\beta {\tilde{M}}_{z}^{2}a_{n}\right]x_{n}=144nNE.$$

Substituting the solution of Equations (19) into (18), we determine the dimensionless modulus $\Delta \tilde{G}$.

## 3. Results

In this part, we compare the results of our calculations with the experiments of [44]. It should be noted that the distribution function *F_N_* over number *N* of particles in the chains has not been determined in [44]. That is why we used the simplest approximation that all chains consist of an identical number *N* of particles. This number has been determined from the condition of the best agreement between the calculated and the measured [44] values of the “no field” modulus $G^{\left(0\right)}$. Some of the results of this comparison and the estimated magnitudes of *N* are given in Table 1.

With respect to the real systems, the estimated *N* can be considered as a characteristic number of the particles per chain.

**Figure 3:**
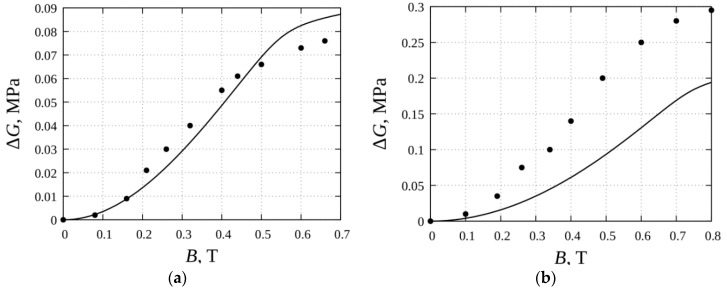
The magnetically induced part *∆G* of the shear modulus vs. the flux density *B* in the composite. Lines—theory, dots—experiment [44]. The shear modulus of the matrix *G*_0_ = 60 kPa; the initial magnetic susceptibility of the particle material *χ_p_* = 100; the saturated magnetization of the particle material *M_s_* = 1670 kA/m; the volume concentration of the particles *φ* = 0.1 (**a**) and *φ* = 0.2 (**b**).

A comparison of our calculations of the magnetically induced part ΔG of the shear modulus *G* with the experiments of [44] are shown in Figure 3.

For the systems with the relatively low volume concentration of the particles (Figure 3a), our results are in good agreement with experiments. Note that any unjustly fit parameters have not been used in our calculation of the modulus ΔG. For the higher concentrations (Figure 3b), the agreement is worse and rather, is only in the frame of the order of magnitude. The physical reason of the worsening of the agreement between the theory and the experiment lies in the fact that besides the linear chains, more topologically complicated branched, net-like, and bulk structures appear in magnetic suspensions with a high concentration of particles [48,49,50,51,52,53,54]. The spatial disposition of the particles is fixed with the host polymer gelation and determines the experimental results for the cured composite. The analysis of morphology of these structures and their effect on the macroscopic properties of the magnetic gels requires a special study. Note that, as a rule, the volume concentration of the particles in ferrogels prepared for biological applications is in the frame of several per cent, or even less than one per cent [9,39,41,66,67]. Figure 3a demonstrates that the present model leads to appropriate results for the low concentrated systems with the internal chains.

**Figure 4:**
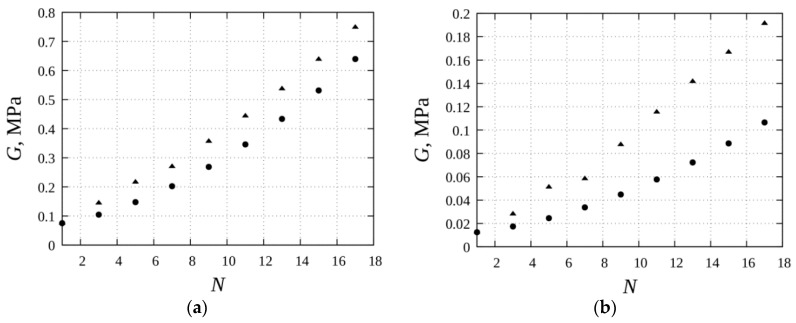
Shear modulus *G* vs. the total number of particles *N.* Squares—magnetic field is zero; triangles—magnetic flux *B* = 0.7 T. Parameters of the system: the initial magnetic susceptibility of the material of the particle material *χ_p_* = 100; the saturated magnetization of the particle material *M_s_* = 1670 kA/m; the volume concentration of the particles *φ* = 0.10. Shear modulus of the elastic matrix *G*_0_ = 60 kPa (**a**) and 10 kPa (**b**).

Our analysis shows that the elastic modulus of the composite significantly depends on the characteristic number *N* of the particles in the chains. The calculated dependencies of *G* on *N* are shown in Figure 4 for the gels, with two different magnitudes of the elastic modulus $G_{0}$ of the polymer matrix.

These results demonstrate that by varying the number *N* of particles in the chains, one can vary in a wide range of magnitudes, the mechanic modulus of the ferrogel. The relative increase of the modulus under the field action is high in soft gels and is less pronounced in the rigid ones. Biological ferrogels, which are used in various applications, are usually soft, with the modulus less than 10 kPa. Therefore, their mechanical properties and behavior can be effectively controlled with the help of an applied magnetic field.

The characteristic length of the chains is determined by the condition of the gel polymerization, which is in part determined by the ratio between the kinetics of the particle’s aggregation and the rate of the host polymer’s curing. Thus, by changing the condition of the system gelation, one can tune in a wide range of the magnitudes of the macroscopic properties of the composite material.

## 4. Discussion

We present the results of the theoretical modeling of magnetorheological effects in magnetic gels with chain-like aggregates. Unlike the previous theoretical models suggested in [44], this model does not contain any unjustly fit parameters. In the frames of applicability of the hypothesis that only linear chains appear at the stage of the composite synthesis (i.e., that only materials with low or moderate concentrations of particles are considered), the model is in good agreement with the experiments of [44] (see Figure 3a). This agreement indicates that the proposed model leads to adequate results for the composite with the concentration of particles, at least in the frames of ten per cent. For the higher concentrations, the appearance of topologically complicated structures is quite probable, and that is why these concentrations are out of the scope of this model.

Note that the volume concentration of the particles in the magnetic gels that are synthesized for biomedical applications, as a rule, is low and in the frames of several per cent (see, for example [9,39,41,66,67] and the references therein). The systems with higher concentrations are usually synthesized for various mechanical systems (dampers, actuators, etc.).

The obtained results can be considered as a theoretical background for the development of technologies of magnetically controllable biosensors, scaffolds with tunable properties, and for engineering and the regeneration of biological tissues.

In real magnetic gels, the distribution over chain size can be quite broad. At the same time, the assumption of the identity of the chains in the composite, which was used in part 2 of this work, is not necessary for the present model. Indeed, the relation 17 and relation 18 can be used to estimate the elastic modulus if the distribution function *F_N_* is known.

Unfortunately, the law of the size of distribution has not been studied in [44]. That is why, and only because of that, that we have used the model of identical chains to compare our results with the experiments [44]. The determined number *N* can be considered as an estimate of the characteristic size of the chains in the real composites.

In principle, our approach allows the studying of the large shear deformations, including the rupture of the chains, and Equations (1)–(3) can be solved numerically in nonlinear approximation with respect to the particles’ displacement *u_i_*. This can be a natural continuation of the present work.

## Figures and Tables

**Table 1:** Comparison between the experimental [44] and our theoretical results for shear modulus $G^{\left(0\right)}$ (no field is applied).

Volume Concentration of the Particles	Experimental [45] Shear Modulus of the Composite without a Magnetic Field (MPa)	Theoretical Shear Modulus of the Composite without a Magnetic Field (MPa)	Estimated Number of Particles *N* in the Chain
10% (V/V) iron	0.26	0.27	9
20% (V/V) iron	0.74	0.81	13

## Appendix A

The coefficients used in relation to (9) for the symmetric stress ${\tilde{\sigma }}_{s}$ read:

$$\alpha_{N}=\frac{1}{N\alpha_{0}\prime },\;\zeta_{N}=\frac{4}{N\left(N^{2}+1\right)\beta_{0}\prime }-\frac{2}{N\alpha_{0}\prime },\;\beta_{N}=\frac{2\left(N^{2}-1\right)}{N\left(N^{2}\alpha_{0}+\beta_{0}\right)},\;\lambda_{N}=\frac{N^{2}-1}{N^{2}+1}.$$

Here,

$$\alpha_{0}=\frac{2}{3},\;N=1,\;\alpha_{0}=-\frac{1}{N^{2}-1}\left[\frac{2}{N}+\frac{\ln\left(2N^{2}-1-2N\sqrt{N^{2}-1}\right)}{\sqrt{N^{2}-1}}\right],\;N>1,\;\beta_{0}=\frac{2}{3},\;N=1,\;\beta_{0}=\frac{1}{N^{2}-1}\left[N-\frac{\ln\left(2N^{2}-1+2N\sqrt{N^{2}-1}\right)}{2\sqrt{N^{2}-1}}\right],\;N>1,\;\alpha_{0}^{\prime }=\frac{2}{5},\;N=1,\;\alpha_{0}^{\prime }=\frac{1}{4{\left(N^{2}-1\right)}^{2}}\left[N\left(2N^{2}-5\right)-\frac{3\ln\left(2N^{2}-1-2N\sqrt{N^{2}-1}\right)}{2\sqrt{N^{2}-1}}\right],\;N>1,\;\beta_{0}^{\prime }=\frac{2}{5},\;N=1,\;\beta_{0}^{\prime }=\frac{1}{{\left(N^{2}-1\right)}^{2}}\left[\frac{N^{2}+2}{N}-\frac{3\ln\left(2N^{2}-1+2N\sqrt{N^{2}-1}\right)}{2\sqrt{N^{2}-1}}\right],\;N>1.$$
